# Subdural empyema complicated after trepanation and drainage of chronic subdural hematoma: A case report

**DOI:** 10.1097/MD.0000000000018587

**Published:** 2019-12-27

**Authors:** Hang Xue, Weitao Zhang, Lin Shi, Yiming Zhang, Bing Yu, Hongfa Yang

**Affiliations:** a Department of Neurotraumatic Surgery, The First Hospital of Jilin University; b Department of Neurosurgery, The Affiliated Hospital of Changchun University of Traditional Chinese Medicine, Changchun, Jilin; c Department of Neurosurgery, Qilu Hospital of Shandong University, Ji’nan, Shandong; d Department of Neurosurgery, Yantai Affiliated Hospital of Binzhou Medical University, Yantai, Shandong, China.

**Keywords:** chronic subdural hematoma, craniotomy, *Klebsiella oxytoca*, subdural empyema

## Abstract

**Rationale:**

Chronic subdural hematoma (CSDH) is one of the most common neurosurgical diseases. However, complicated subdural empyema rarely occurs after trepanation and drainage of chronic subdural hematoma.

**Patient concerns:**

A male patient (77 years old) was admitted to the hospital on the 2nd day of fever after an undergoing a “trepanation and drainage of chronic subdural hematoma” operation at a local hospital. After admission, the patient was treated with an emergency operation in which a subdural abscess was diagnosed and then administered antibiotics after the operation.

**Diagnosis:**

According to the clinical manifestations, intraoperative findings of imaging examination and the results of pus culture, the diagnosis was subdural empyema.

**Intervention:**

We surgically removed the subdural empyema. Postoperative antibiotics were administered according to the results of bacterial culture.

**Outcomes:**

At 3 months after the operation, the patient returned to the hospital for reexamination and was found to have achieved a good recovery and good self-care.

**Lessons:**

Subdural empyema after trepanation and drainage of chronic subdural hematoma is a very rare and severe disease. Early diagnosis and operative intervention as well as the intravenous administration of antibiotics can improve the prognosis of patients and enhance their quality of life.

## 1 Introduction

Chronic subdural hematoma (CSDH) is one of the most common neurosurgical diseases.[^1^,^2^,^3^,^4^,^5^,^6^,^7^] An operation for trepanation and drainage of chronic subdural hematoma is a mature approach. It was first reported by McKissock and Richardson in 1960s.^[4]^ Currently, it is the most widely used surgical method for the treatment of CSDH. And Weigel et al indicate that this type of surgery is the most efficient choice for surgical drainage of uncomplicated CSDH.[^4^,^5^,^6^]; complications are very rare after an operation for subdural empyema.[^2^,^3^] In this study, we discuss a case of subdural empyema caused by infection with *Klebsiella oxytoca* after trepanation and drainage of chronic subdural hematoma. After we contacted the ethics committee of The First Hospital of Jilin University, ethical approval was not required for this case report article. Informed consent was obtained from the patient for the publication of this case report.

## 2 Case report

**Figure 1:**
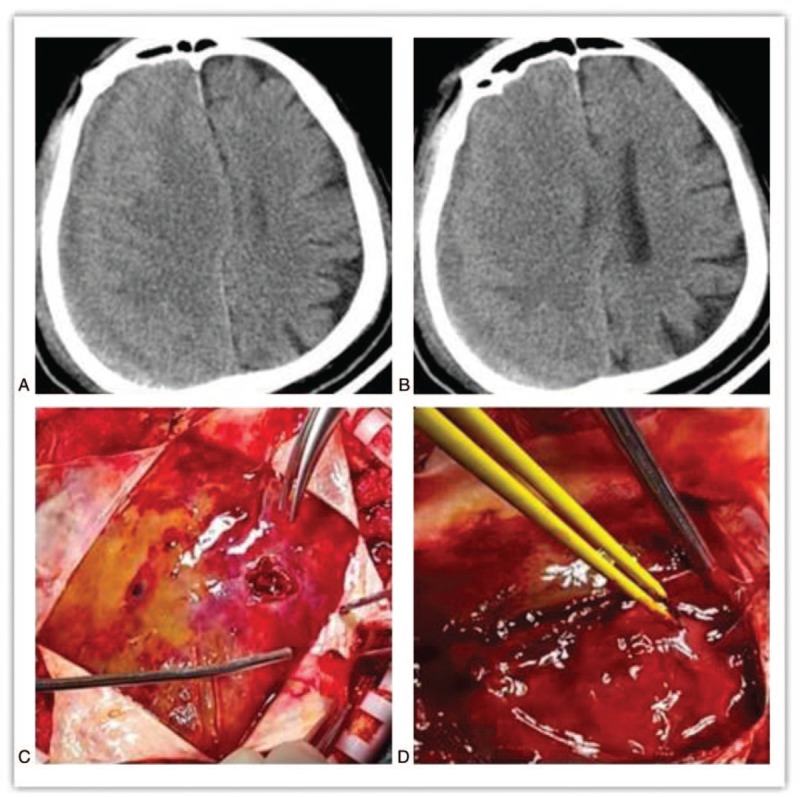
Findings during preoperative CT are shown in A and B: slightly high bands were observed below the right frontotemporal top plates with a low-density shape, compressed right brain tissues, and a shallow cerebral sulcus that then disappeared. The right ventricle was compressed and decreased in size. The median line was shifted toward the left side. Intraoperative images are shown in C and D: A large amount of yellow-white purulent fluid and bloody fluid were found subdurally of the frontotemporal region, which showed a complete capsule and obvious local separation.

A male patient (77 years old) presented 3 days after an operation for “trepanation and drainage of chronic subdural hematoma” at a local hospital. The patient run a fever for 2 days and was admitted to the hospital on July 15, 2018. In his past medical history, the patient had suffered from diabetes for 25 years and had achieved mediocre effect by controlling his blood glucose levels with insulin. Admission PE: His Glasgow Coma Scale score was E3V5M6. On the right side of the wound, redness and swelling were observed at 1 operative incision, and there was a pink purulent secretion spilling out of the incision. The bilateral pupils were 2.0 mm in diameter and showed slow direct and indirect reactions to light. The limbs showed low muscle tension. The muscle strength of the left upper limbs was grade I, while that of the lower limbs was grade II, and that of the right limbs was grade V. Hyperreflexia was observed in the deep and superficial reflex in the right limbs, and the Babinski sign was positive. The patient's neck was stiff at a position of 3 transverse fingers. Findings on cranial CT showed a localized defect of the right parietal bone, a low density area in the right subdural space of the frontal, parietal, and temporal regions. The right brain tissues were compressed, and the cerebral sulcus was shallow and had disappeared. The right ventricle was compressed and decreased in size. The median line had shifted toward the left side (Fig. 1 A, B).

**Figure 2:**
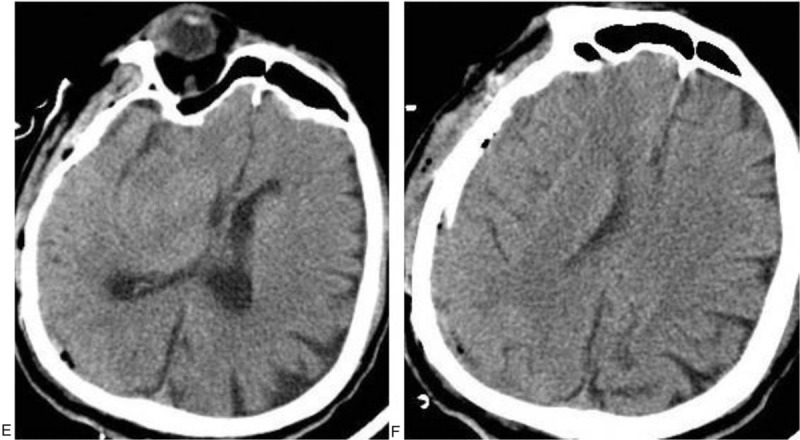
Postoperative reexamination by CT showed that the slightly higher bands below the right frontotemporal top plates and the low-density shape had disappeared, the right brain tissues were relieved from compression, and the sulcus had reappeared. The median line had returned to a normal position.

During the operation, a drainage tube was placed subdurally through the original drill hole, and a large amount of purulent liquid and a small amount of bloody liquid were collected. The patient was diagnosed with “subdural empyema”, and craniotomy was performed. The dura mater was cut open at the site of the defect, a large amount of purulent yellow-white fluid was found subdurally in the frontotemporal part of the wound, and local separation had occurred (Fig. 1C, D). The subdural abscess was removed, and the pus was collected for culture. After the operation, the patient's temperature essentially returned to normal with an occasional low fever. The results of pus culture indicated “*Klebsiella oxytoca*” (Table 1). Third-generation cephalosporins was combined with linezolid and administered for treatment. The patient was discharged 35 days after admission. A postoperative CT examination showed the patient had achieved a good recovery (Fig. 2 E, F). When discharged, the patient had good mental status and was able to answer simple questions correctly with occasional speech disorder. Normal muscular tension was observed in the limbs, muscle strength was grade IV, and the mobility of the left limbs was poorer than that of the right limbs. At 3 months after the operation, the patient's general state had recovered with self-care.

**Table 1:**
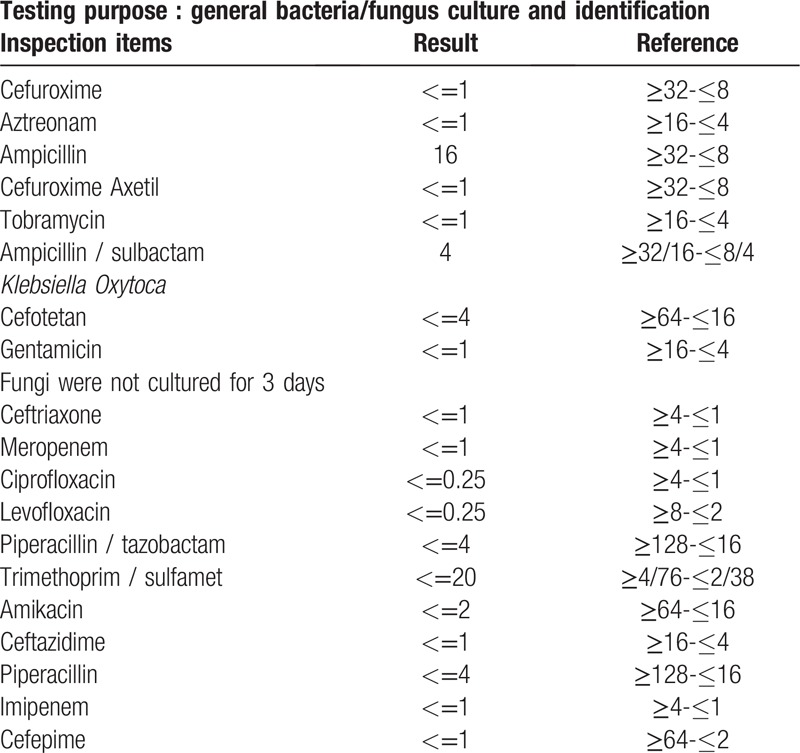
Intraoperative extraction of secretion cultures suggested infection with *Klebsiella oxytoca*.

## 3 Discussion

Chronic subdural hematoma is one of the most common neurosurgical diseases.[^1^,^2^,^3^,^4^,^5^,^6^,^7^] An operation for trepanation and drainage of chronic subdural hematoma is a mature approach. It was first reported by McKissock and Richardson in 1960s.^[4]^ Currently, it is the most widely used surgical method for the treatment of CSDH. And Weigel et al indicate that this type of surgery is the most efficient choice for surgical drainage of uncomplicated CSDH.[^4^,^5^,^6^] Subdural empyema is an extremely rare complication after this operation.[^2^,^8^,^9^] According to the relevant available data, subdural empyema is mainly observed in open craniocerebral injuries, craniocerebral operations, and chronic systemic infectious diseases.[^9^,^10^,^11^,^12^] In 2003, Choi et al reported a chronic subdural hematoma infection caused by a hepatic abscess^[9]^; and in 1998, Kawamoto et al reported a case of subdural hematoma infection caused by a thigh infection^[13]^ and diabetes as well as in tumors or immunodeficiency diseases. Chan et al reported 1 case of chronic subdural hematoma infection caused by radiotherapy and chemotherapy for nasopharynx cancer.^[14]^ However, subdural empyema has a relatively high mortality rate (7%–30%).[^2^,^8^,^9^] The most common infectious pathogen is *Streptococcus milleri* group, which is followed by other pathogenic bacteria, including *β-hemolytic Streptococcus*, *Staphylococcus aureus*, *Haemophilus influenzae*, *Streptococcus mutans*, and *Pseudomonas*. *Escherichia coli*,^[15]^ *Propionibacterium acnes*,^[16]^ *Campylobacter fetus* ^[17]^ and *Klebsiella*,[^9^,^18^] which are more rarely observed pathogens. Overall, 20% to 30% of cases of subdural empyema are not caused by infection with a single bacteria.[^8^,^9^,^15^,^16^,^17^,^18^] In this case, the patient was infected with *Klebsiella*, a gram-negative bacillus that generally occurs in immunocompromised hosts.^[9]^ This patient had a 25-year history of diabetes, which is a high risk factor for infection. In addition, the side effects of the craniocerebral operation and the abundant vascular beds caused by chronic subdural hematoma provided a favorable culture medium for bacteria and are important factors that can cause complication with subdural empyema. Clinically, the infected subdural hematoma manifests as nonspecific infection symptoms, focal neurological signs, and increased intracranial pressure.^[19]^ Patients with these clinical manifestations are highly suspected of subdural empyema, and it is necessary to perform imaging examinations. CT scanning will show the subdural empyema as a thin layer of liquid characterized by edge enhancement that has a slightly higher density than cerebrospinal fluid, adjacent brain tissues will show edema to various extents, and the sulci will have disappeared.[^20^,^21^] When the state of illness permits, magnetic resonance imaging (MRI) examination is required; compared with CT scanning, MRI can show additional axial flow and edge enhancement and can visualize the presence of pus.[^22^,^23^] The critical determining factor that influences the prognosis of patients with subdural empyema is the active and early removal of the source of infection, the drainage of subdural pus and appropriate treatment of the infection with antibiotics.^[3]^ For patients with subdural empyema, various operative approaches, such as conservative treatment with drugs, craniotomy, and extracranial drainage, have been discussed. Early scholars, such as Dill et al, proposed that operative approaches had little influence on clinical outcomes when the operation was performed as soon as possible and the abscess removed as clearly as possible^[24]^; however, in recent years, most scholars believe that compared with the drainage approach, craniotomy has lower mortality and a lower recurrence rate, and most scholars therefore advocate treatment by craniotomy.[^25^,^26^,^27^,^28^] However, not all patients should be treated with a conventional operation. In the event that a patient has small neurological impairment, shows no obvious abscess on CT scanning and shows rapidly improved clinical manifestations after treatment with antibiotics, conservative treatment with drugs can be chosen as long as continuous MRI monitoring shows a gradually dissipated lesion.^[29]^ In the current case, the patient showed significant symptoms of infection in addition to neurological defects, and CT showed obvious subdural empyema and swelling of brain tissues under compression. Therefore, a large craniotomy was performed to completely remove the subdural empyema, and the necessity of decompressive craniectomy was judged according to the bleeding observed in cortical regions and the compression of brain tissues after the abscess was removed.

## Author contributions

**Conceptualization:** Hongfa Yang.

**Data curation:** Hang Xue, Weitao Zhang, Hongfa Yang.

**Funding acquisition:** Hongfa Yang.

**Investigation:** Weitao Zhang, Lin Shi, Yiming Zhang.

**Resources:** Weitao Zhang, Bing Yu, Hongfa Yang.

**Software:** Lin Shi.

**Visualization:** Lin Shi.

**Writing – original draft:** Hang Xue.

**Writing – review & editing:** Hang Xue.
